# The impact of low‐dose CT on smoking behavior among non‐smokers, former‐smokers, and smokers: A population‐based screening cohort in rural China

**DOI:** 10.1002/cam4.5073

**Published:** 2022-07-27

**Authors:** Zheng Su, Xuebing Li, Heng Wu, Zhaowei Meng, Yang Li, Hongli Pan, Hao Liang, Ying Wang, Fang‐Hui Zhao, Youlin Qiao, Qinghua Zhou, Ya‐Guang Fan

**Affiliations:** ^1^ Department of Cancer Epidemiology, National Cancer Center/Cancer Hospital Chinese Academy of Medical Sciences and Peking Union Medical College Beijing China; ^2^ Tianjin Key Laboratory of Lung Cancer Metastasis and Tumor Microenvironment, Tianjin Lung Cancer Institute Tianjin Medical University General Hospital Tianjin China; ^3^ Department of Nuclear Medicine Tianjin Medical University General Hospital Tianjin China; ^4^ Sichuan Lung Cancer Institute, Sichuan Lung Cancer Center, West China Hospital, Chengdu Sichuan University China; ^5^ Department of Radiology Tianjin Medical University General Hospital Tianjin China; ^6^ Center of Global Health, School of Population Medicine and Public Health Chinese Academy of Medical Sciences and Peking Union Medical College Beijing China

**Keywords:** low‐dose computed tomography, lung cancer screening, smoking behavior

## Abstract

**Background:**

Lung cancer screening may provide a “teachable moment” for the smoking cessation and relapse prevention. However, the impact of lung cancer screening on smoking initiation in non‐smokers has not been reported.

**Methods:**

A baseline smoking behavior survey was conducted in 2000 participants who were screened by low‐dose computed tomography (LDCT) from 2014 to 2018. All participants were re‐surveyed on their smoking behavior in 2019. Of these, 312 participants were excluded, leaving 1688 participants in the final analysis. The smoking initiation rate in baseline non‐smokers, the relapse rate in baseline former smokers, and the abstinence rate in baseline current smokers were calculated, respectively. The associations between screening results, demographic characteristics, and smoking behavior change were analyzed using multivariable logistic regression.

**Results:**

From 2014 to 2019, smoking prevalence significantly decreased from 52.6% to 49.1%. The prevalence of smoking initiation, relapse, and abstinence in baseline non‐smokers, former, and current smokers was 16.8%, 22.9%, and 23.7%, respectively. The risk of smoking initiation in baseline non‐smokers was significantly higher in those with negative screening result (adjusted OR = 2.97, 95% CI: 1.27–6.94). Compared to smokers who only received baseline screening, the chance of smoking abstinence in baseline current smokers was reduced by over 80% in those who attended 5 rounds of screening (adjusted OR = 0.15, 95% CI:0.08–0.27). No significant associations were found between smoking relapse and prior screening frequency, with at least one positive screening result. Age, gender, occupational exposure, income, and smoking pack years were also associated with smoking behavior changes.

**Conclusions:**

The overall decreased smoking prevalence indicated an overwhelming effect of “teachable moment” on “license to smoke.” A tailored smoking cessation strategy should be integrated into lung cancer screening.

## Graphical abstract

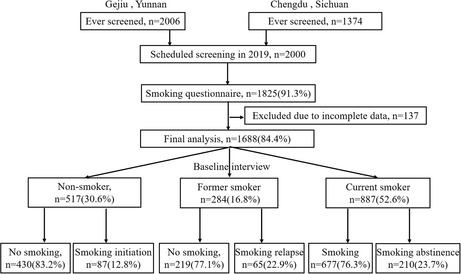

## 1 INTRODUCTION

In China, lung cancer has become the leading cancer‐related death since the beginning of this century.^1^ Tobacco smoking accounted for 75.0% and 18.4% of lung cancer deaths for men and women, respectively, in China.^2^ Numerous case–control and cohort studies have demonstrated a substantial reduction in lung cancer risk in former smokers compared with current smokers.^3^, ^4^ Accordingly, tobacco control is the principal approach to the primary prevention of lung cancer.^5^ As to secondary prevention, randomized controlled trials reported the effectiveness of low‐dose helical computed tomography CT (LDCT) screening of lung cancer.^6^, ^7^ Subsequently, many medical organizations recommended LDCT screening in high‐risk populations, especially heavy smokers.

Current lung cancer screening guidelines recommend the integration of smoking cessation practices into lung cancer screening.^8^, ^9^ The combination of lung cancer screening and smoking cessation was reported to be more effective in reducing mortality than either LDCT lung cancer screening or smoking cessation alone.^10^, ^11^ Lung cancer screening may also represent a teachable moment and an opportunity to enhance motivation for smoking abstinence, especially among those who receive a positive scan result.^12^, ^13^, ^14^, ^15^ However, some other studies found no significant impact of lung cancer screening on smoking cessation, even an adverse impact.^16^ Similarly, the relationship between lung cancer screening and smoking relapse was also inconsistent. Furthermore, there is a concern that lung cancer screening might give some participants an unrealistic feeling of reassurance, which leads to continued smoking or smoking relapse (license to smoke), especially in those who receive negative screening results.^12^, ^17^

The impact of lung cancer screening on smoking initiation in non‐smokers has not been reported, since most lung cancer screening trials/studies have been conducted in heavy smokers. However, due to the special risk factor spectrum of lung cancer in Asia, especially in China, several randomized controlled trials and observational studies recruited non‐smokers who had other risk factors including prior lung disease, second‐hand smoking, occupational, or household cooking fume exposure to receive LDCT lung cancer screening.^18^, ^19^, ^20^ This provided an opportunity to explore the impact of LDCT lung cancer screening on the smoking status of non‐smokers. In 2009, a government‐sponsored multiple‐center lung cancer screening program in rural China (LungSPRC) was initiated. In this project, some non‐smokers but with other lung cancer risk factors were enrolled. In 2019, we conducted a smoking survey in two screening centers of LungSPRC with the aim to investigate the impact of LDCT screening on the smoking behaviors of baseline non‐smokers, former, and current smokers.

## 2 MATERIALS AND METHODS

### 2.1 Study design and population

**FIGURE 1:**
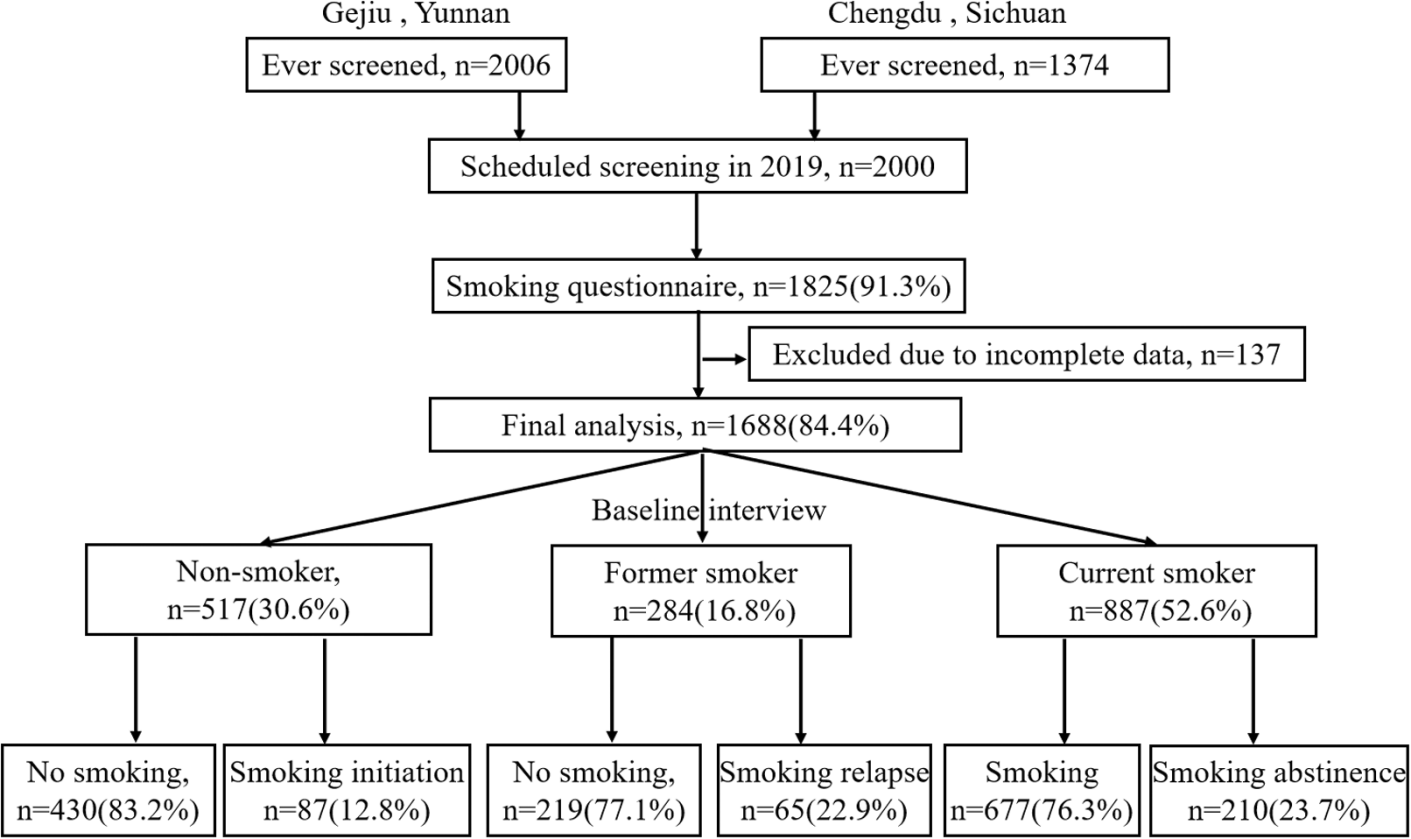
Flow chart of selections of the participants included in the final analysis.

Our study is a multi‐cross‐sectional study. All 2000 participants from Yunnan and Sichuan provinces were surveyed for smoking behavior in 2014 and 2019, respectively. From April 2014 to December 2018, a total of 5 rounds of low‐dose computed tomography (LDCT) screening were conducted on this population. Finally, 312 participants were excluded, and 1688 participants were included in the final analysis. More details are shown in Figure 1.

The criteria for a high‐risk population in LungSPRC are defined as follows: (i) Participants were 40–74 years old with occupational exposure, or 50–74 years old without occupational exposure; (ii) at least 20 pack‐years smoking history, and, if former smokers, had quit within the previous 5 years; (iii) having a history of 10 or more years of underground mining and/or smelting experience. Participants who satisfied the criteria (i) and (ii), or (i) and (iii), or (i), (ii), (iii) were considered high‐risk. The exclusion criteria of this screening program are defined as follows: (i) A proven history of previous malignancy within 5 years (except non‐melanoma skin cancer, cervical cancer in situ, and localized prostate cancer), (ii) individuals who cannot tolerate possible lung cancer resection or who have serious life‐threatening illnesses were not recommended to screened by LDCT.^21^

### 2.2 Baseline information

Detailed information about demographic characteristics, tobacco consumption, medical history, and occupational exposure was collected with a standardized questionnaire at the baseline interview. The participant was considered to have occupational exposure if he or she had ever been exposed to carcinogens including arsenic, asbestos, chromate compound, coke oven emissions, arsenic, or chloromethyl ether. Prior lung disease was defined as the existence of asthma, chronic bronchitis, emphysema, silicosis, tuberculosis, or chronic obstructive pulmonary disease.

Information on age of start/stop smoking, type of tobacco (cigarette, waterpipe, long‐stem pipe), and smoking status was collected at baseline. For smoking status, individuals who had smoked cigarettes regularly for 6 months or longer were defined as baseline smokers, while those who had a smoking duration of less than 6 months in their lifetime before the baseline interview were considered baseline non‐smokers. Pack‐year was calculated by multiplying the number of packs of cigarettes smoked per day by the number of years the person had been smoking.

### 2.3 LDCT lung cancer screening

Participants were invited to undergo baseline LDCT screening and the following annual screening.^22^, ^23^ LDCT screening was performed in accordance with the China national lung cancer screening guideline, which was developed by the China lung cancer early detection and treatment expert group. Detailed information about scan parameters, image observation, nodule measurements nodule management, and follow‐up can be seen elsewhere.^24^ In baseline screening, participants were considered positive with solid or part‐solid nodules ≥5 mm in diameter, nonsolid nodules ≥8 mm in diameter, or airway lesions, nodules, and masses suspicious of lung cancer. In annual screening, a positive lesion was defined as any new non‐calcified nodule or new airway lesion, enlarged nodule, or nodule with an increase in solid component compared to the previous scan. Participants with positive scans will be followed up according to the properties and size of the nodules. In the LungSPRC project, no specific tobacco control measure was provided to participants.

### 2.4 Smoking behavior survey

At the beginning of 2019, we conducted a smoking behavior survey just before the annual screening this year. A total of 2000 participants who would receive the upcoming screening were invited to fill a smoking behavior questionnaire. Respondents who answered that they still out smoked were classified as current smokers. Then, these participants were asked questions related to their smoking intensity (number of cigarettes smoked per day), nicotine dependency, and motivation to quit smoking. Respondents who answered that they did not smoke at the survey were asked the following questions: (1) Date of quitting, (2) Have you quitted smoking for 6 months now(no/yes)? (3) Have you smoked since you quit smoking (no/1–5 cigarette/>5 cigarette)? (4) What is the reason for your quitting smoking?

Nicotine dependence and motivation to quit smoking in current smokers and relapsed smokers were also evaluated. The first question in the Fagerström Questionnaire is “How soon after you wake up do you smoke your first cigarette?” (0 = 61 min or more/1 = 31–60 min/2 = 6–30 min/3 = within 5 min). A high rating implies a high nicotine dependence. Motivation to quit smoking was investigated using the question “Do you have any plans to quit smoking?” (not at all/Yes, but no specific smoking cessation plan/Will quit smoking within 6–12 months/Will quit within 1–6 months/Will quit within 1 month).

### 2.5 Statistical analysis

Based on the results of the baseline and this survey, we defined status changed from never smoking, former smoking at baseline to current smoking as smoking initiation or smoking relapse, respectively. Smoking abstinence was defined as a report of no smoking in those who were current smokers at baseline. Besides, point prevalence abstinence was defined as the report of not currently smoking at this survey in baseline current smokers; sustained abstinence was defined as a report of not currently smoking at this survey, and no cigarettes smoked in the past 6 months.

In our study, the prevalence of smoking initiation, smoking abstinence, and smoking relapse was the primary outcome measure. The difference in smoking prevalence between baseline and the current survey was compared by the McNemar chi‐square test. The differences in baseline characteristics according to the current smoking status in baseline non‐smokers, former smokers, and current smokers were compared using the chi‐square test or Fisher's exact test appropriately. Multivariable (backward) logistic regression analyses were performed to investigate whether baseline characteristics and prior screening results can predict smoking initiation, smoking relapse, and smoking abstinence in baseline non‐, former smokers, and current smokers, respectively. Statistical analysis was performed using Stata 14.0 software, and a *p*‐value <0.05 was considered statistically significant.

## 3 RESULTS

Among 1688 participants, the proportions of baseline non‐smoker, former and current smokers were 30.6%,16.8%, and 52.6% (Table 1). The smoking initiation rate in baseline non‐smokers was 16.8%, the relapse rate in baseline former smokers was 22.9%, and the abstinence rate in baseline current smokers was 23.7%, respectively.

**TABLE 1:** Smoking status and screening results according to baseline smoking status

Smoking prevalence and Screening status	Baseline smoking status			
	None (*n* = 517,30.6%)	Former (*n* = 284,16.8%)	Current (*n* = 887,52.6%)	*p*
Smoking status at 2019				
No	430 (83.2)	219 (77.1)	210 (23.7)	<0.001
Yes	87 (16.8)	65 (22.9)	677 (76.3)	
Age				
<50	74 (14.3)	3 (1.1)	41 (4.6)	<0.001
50–59	319 (61.7)	102 (35.9)	419 (47.2)	
60–70	117 (22.6)	158 (55.6)	383 (43.2)	
70	7 (1.4)	21 (7.4)	44 (5.0)	
Gender				
Male	128 (24.8)	264 (93.0)	875 (98.7)	<0.001
Female	389 (75.2)	20 (7.0)	12 (1.4)	
Marriage				
Married	456 (88.2)	275 (96.8)	839 (94.6)	<0.001
Others	61 (11.6)	48 (5.4)	48 (5.4)	
Annual household income (¥)				
<20,000	143 (27.7)	58 (20.4)	186 (21.0)	<0.001
20,000‐29,999	74 (14.3)	52 (18.3)	197 (22.3)	
30,000‐49,999	239 (46.2)	107 (37.7)	303 (34.3)	
50,000‐	61 (11.8)	67 (23.6)	198 (22.4)	
Education				
Primary school or less	141 (27.3)	102 (35.9)	333 (37.5)	<0.001
Junior high/high school	370 (71.6)	146 (51.4)	481 (54.2)	
College/above	6 (1.2)	36 (12.7)	73 (8.2)	
Occupational exposure				
No	113 (21.9)	194 (68.3)	609 (68.7)	<0.001
Yes	404 (78.1)	90 (31.7)	278 (31.4)	
Prior lung disease				
No	501 (96.9)	232 (81.7)	800 (90.2)	<0.001
Yes	16 (3.1)	52 (18.3)	87 (9.81)	
Screening number				
1	41 (7.9)	10 (3.5)	67 (7.6)	<0.001
2	91 (17.6)	49 (17.3)	116 (13.1)	
3	255 (49.3)	72 (25.4)	210 (23.7)	
4	111 (21.5)	35 (12.3)	143 (16.1)	
5	19 (3.7)	118 (41.6)	351 (39.6)	
Positive screens				
No	410 (79.3)	227 (79.9)	699 (78.8)	0.916
At least one	107 (20.7)	57 (20.1)	188 (21.2)	

Table 1 shows the personal characteristics, prior screening frequency and positivity history of baseline non‐smokers, former and current smokers. Compared to former and current smokers, baseline non‐smokers were more females, had lower age, income level, and fewer prior screening numbers, but higher occupational exposure. In addition, no significant difference in prior positive screening history was observed among these three groups.

Among 517 baseline non‐smokers, 87 (16.8%) reported a smoking initiation in this survey. In the univariable analysis (Table 2), compared to those continued non‐smokers, new smokers were more common among males, younger participants, and participants with lower education levels. The distributions of occupational exposure and prior screening frequency were also significantly different between these two groups. The result of multivariable logistic regression analysis (Table 3) suggested that the risk of smoking initiation in baseline non‐smokers was significantly lower in females, with an adjusted OR of 0.003 (95% CI: 0.001–0.011). Besides, this risk was significantly decreased with age, and the adjusted ORs for those aged 50–59 and 60–69 compared to those aged <50 were 0.12 (95% CI: 0.01–1.22) and 0.09 (95% CI: 0.01–1.01), respectively. In contrast, pure negative screening results significantly increased the risk of smoking initiation (adjusted OR = 2.97, 95% CI: 1.27–6.94).

**TABLE 2:** Odd ratios and 95% CIs of smoking behaviors by univariate logistic regression analysis

Characteristics	Smoking Initiation in nonsmokers			Point smoking in former smokers			Point cessation in smokers			Sustained cessation in smokers		
	No	Yes	OR (95% CI)	No	Yes	OR (95% CI)	Yes	No	OR (95% CI)	Yes	No	OR (95% CI)
Age												
<50	69 (16.1)	5 (5.8)	1	1 (33.3)	2 (66.7)	1	37 (90.2)	4 (9.8)	1	37 (90.2)	4 (9.8)	1
50–59	275 (64.0)	44 (50.6)	2.21 (0.84–5.78)	64 (62.8)	38 (37.3)	0.30 (0.03–3.38)	330 (78.8)	89 (21.2)	2.49 (0.87–7.18)	330 (81.5)	75 (18.5)	2.10 (0.73–6.08)
60–70	80 (18.6)	37 (42.5)	6.38 (2.37–17.13)	135 (85.4)	23 (14.6)	0.09 (0.01–0.99)	278 (72.6)	105 (27.4)	3.49 (1.22–10.04)	278 (78.1)	78 (21.9)	2.60 (0.90–7.50)
70‐	6 (1.4)	1 (1.2)	2.30 (0.23–23.02)	19 (90.5)	2 (9.5)	0.05 (0.00–0.87)	32 (72.7)	12 (27.3)	3.47 (1.02–11.83)	32 (76.2)	10 (23.8)	2.89 (0.83–10.11)
Gender												
Male	44 (89.8)	84 (96.6)	1	201 (76.1)	63 (23.9)	1	673 (76.9)	202 (23.1)	1	673 (80.9)	159 (19.1)	1
Female	386 (10.2)	3 (3.5)	0.004 (0.001–0.01)	18 (90.0)	2 (10.0)	0.35 (0.08–1.57)	4 (33.3)	8 (66.7)	6.66 (1.98–22.35)	4 (33.3)	8 (66.7)	8.47 (2.52–28.46)
Marriage												
Married	375 (87.2)	81 (93.1)	1	211 (76.7)	64 (22.3)	1	643 (76.6)	196 (23.4)	1	643 (80.6)	155 (19.4)	1
Others	55 (12.8)	6 (6.9)	0.50 (0.21–1.21)	8 (88.9)	1 (11.1)	0.41 (0.05–3.36)	34 (70.8)	14 (29.2)	1.35 (0.71–2.57)	34 (73.9)	12 (26.1)	1.46 (0.74–2.89)
Annual household income (¥)												
<20,000	114 (26.5)	29 (33.3)	1	46 (79.3)	12 (20.7)	1	136 (73.1)	50 (26.9)	1	136 (76.8)	41 (23.2)	1
20,000‐29,999	57 (13.3)	17 (19.5)	1.17 (0.60–2.31)	42 (80.8)	10 (19.2)	0.91 (0.36–2.33)	147 (74.6)	50 (25.4)	0.93 (0.59–1.46)	147 (79.5)	38 (20.5)	0.86 (0.52–1.41)
30,000‐49,999	214 (49.8)	25 (28.7)	0.46 (0.26–0.82)	75 (70.1)	32 (29.9)	1.64 (0.77–3.49)	226 (74.6)	77 (25.4)	0.93 (0.61–1.40)	226 (78.2)	63 (21.8)	0.92 (0.59–1.45)
50,000‐	45 (10.5)	16 (18.4)	1.40 (0.69–2.82)	56 (83.6)	11 (16.4)	0.75 (0.30–1.86)	166 (83.8)	32 (16.2)	0.52 (0.32–0.86)	166 (86.9)	25 (13.1)	0.50 (0.29–0.43)
Education												
Primary school or less	95 (22.1)	46 (52.9)	1	71 (69.6)	31 (30.4)	1	255 (76.7)	78 (23.4)	1	255 (81.0)	60 (19.1)	1
Junior high/high school	330 (76.7)	41 (46.0)	0.25 (0.15–0.41)	115 (78.8)	31 (21.2)	0.62 (0.35–1.10)	366 (76.1)	115 (23.9)	1.02 (0.74–1.43)	366 (79.7)	93 (20.3)	1.08 (0.75–1.55)
College/above	5 (1.2)	1 (1.2)	0.41 (0.05–3.64)	33 (91.7)	3 (8.3)	0.21 (0.06–0.73)	56 (76.7)	17 (23.3)	0.99 (0.55–1.81)	56 (80.0)	14 (20.0)	1.06 (0.55–2.03)
Occupational exposure												
No	82 (19.1)	31 (35.6)	0.001	179 (92.3)	15 (7.7)	1	487 (80.0)	122 (20.0)	1	487 (84.7)	88 (15.3)	1
Yes	348 (80.9)	56 (64.4)	0.43 (0.26–0.70)	40 (44.4)	50 (55.6)	14.92 (7.62–29.18)	190 (68.4)	88 (31.7)	1.84 (1.34–2.55)	190 (70.6)	79 (29.4)	2.30 (1.63–3.26)
Prior lung disease												
No	419 (97.4)	82 (94.3)	1	177 (76.3)	55 (23.7)	1	616 (77.0)	184 (23.0)	1	616 (80.7)	147 (19.3)	1
Yes	11 (2.6)	5 (5.8)	2.32 (0.79–6.86)	42 (80.8)	10 (19.2)	0.77 (0.36–1.63)	61 (70.1)	26 (29.9)	1.43 (0.88–2.32)	61 (75.3)	20 (24.7)	1.37 (0.80–2.35)
Prior screening number												
1	35 (8.1)	6 (6.9)	1	8 (80.0)	2 (20.0)	1	36 (53.7)	31 (46.3)	1	36 (59.0)	25 (41.0)	1
2	77 (17.9)	14 (16.1)	1.06 (0.38–2.99)	32 (65.3)	17 (34.7)	2.12 (0.41–11.14)	82 (70.7)	34 (29.3)	1.00 (0.44–2.24)	82 (72.6)	31 (27.4)	1.17 (0.50–2.77)
3	216 (50.2)	39 (44.8)	1.05 (0.42–2.67)	43 (59.7)	29 (40.3)	2.70 (0.53–13.62)	143 (68.1)	67 (31.9)	1.56 (0.75–3.24)	143 (73.0)	51 (27.0)	1.57 (0.71–3.46)
4	95 (22.1)	16 (18.4)	0.98 (0.35–2.71)	28 (80.0)	7 (20.0)	1.00 (0.17–5.79)	108 (75.2)	35 (24.5)	1.09 (0.50–2.36)	108 (79.4)	28 (20.6)	1.15 (0.50–2.64)
5	7 (1.6)	12 (13.4)	10.00 (2.80–35.69)	108 (91.5)	10 (8.5)	0.37 (0.07–1.99)	308 (87.8)	43 (12.3)	0.74 (0.36–1.53)	308 (91.1)	30 (8.9)	0.61 (0.28–1.35)
Positive screening result												
No	339 (78.8)	71 (81.6)	1	174 (76.7)	53 (23.4)	1	530 (75.8)	169 (24.2)	1	530 (80.1)	132 (19.9)	1
At least one	91 (21.2)	16 (18.4)	0.84 (0.47–1.51)	45 (79.0)	12 (21.1)	0.87 (0.43–1.78)	147 (78.2)	41 (21.8)	1.07 (0.74–1.55)	147 (80.8)	35 (19.2)	1.10 (0.73–1.64)
Pack‐year												
<20	—	—	—	50 (61.0)	32 (39.0)	1	102 (64.2)	57 (35.9)	1	102 (67.6)	49 (32.5)	1
20–39	—	—	—	122 (82.4)	26 (17.6)	0.33 (0.18–0.61)	352 (79.1)	93 (20.9)	0.47 (0.33–0.70)	352 (83.0)	72 (17.0)	0.43 (0.28–0.65)
40‐	—	—	—	47 (87.0)	7 (13.0)	0.23 (0.09–0.58)	223 (79.1)	59 (20.9)	0.48 (0.31–0.74)	223 (82.9)	46 (17.1)	0.43 (0.27–0.68)
Quitting years												
0–4	—	—	—	165 (75.3)	32 (49.2)	1	—	—	—	—	—	—
5–10	—	—	—	9 (4.1)	7 (10.8)	4.01 (1.39–11.55)	—	—	—	—	—	—
>10	—	—	—	8 (3.7)	12 (18.5)	7.73 (2.93–20.43)	—	—	—	—	—	—
Unknown	—	—	—	37 (16.9)	14 (21.5)	1.95 (0.95–4.02)	—	—	—	—	—	—

**TABLE 3:** Odd ratios and 95% CIs of smoking behaviors by multivariate logistic regression analysis^a^

Characteristics	Smoking Initiation in nonsmokers	Point smoking in former smokers	Point cessation in smokers	Sustained cessation in smokers
Age				
<50	Reference	Reference	Reference	Reference
50–59	0.13 (0.01–1.45)	0.24 (0.06–0.88)	4.15 (1.11–15.46)	3.84 (0.97–15.23)
60–70	0.09 (0.01–1.07)	0.17 (0.04–0.61)	5.90 (1.59–21.90)	4.63 (1.17–18.29)
70‐	0.01 (0.00–0.23)	0.12 (0.03–0.53)	8.21 (1.87–36.04)	7.94 (1.67–37.75)
Gender				
Male	Reference	Reference	Reference	Reference
Female	0.003 (0.001–0.011)	0.41 (0.07–2.32)	7.23 (1.82–28.67)	9.33 (2.36–36.92)
Annual household income(¥)				
<20,000	Reference	Reference	Reference	Reference
20,000–29,999	0.99 (0.31–3.20)	1.02 (0.30–3.46)	0.84 (0.51–1.39)	0.72 (0.41–1.26)
30,000–49,999	0.50 (0.18–1.37)	0.66 (0.24–1.82)	0.85 (0.54–1.36)	0.78 (0.47–1.30)
50,000‐	0.81 (0.24–2.72)	0.77 (0.24–2.42)	0.52 (0.30–0.88)	0.47 (0.26–0.85)
Occupational exposure				
No	Reference	Reference	Reference	Reference
Yes	0.50 (0.15–1.65)	11.68 (4.81–28.38)	1.08 (0.72–1.62)	1.41 (0.91–2.18)
Prior lung disease				
No	Reference	Reference	Reference	Reference
Yes	0.66 (0.13–3.32)	1.07 (0.41–2.82)	1.78 (1.03–3.05)	1.89 (1.04–3.45)
Pack‐year				
<20	—	Reference	Reference	Reference
20–39	—	0.61 (0.27–1.34)	0.59 (0.38–0.90)	0.56 (0.35–0.89)
40‐	—	0.39 (0.13–1.19)	0.56 (0.35–0.89)	0.52 (0.31–0.86)
Prior screening number				
1	Reference	Reference	Reference	Reference
2	1.03 (0.19–5.61)	3.08 (0.45–20.95)	0.46 (0.24–0.88)	0.50 (0.25–0.97)
3	0.91 (0.19–4.22)	3.56 (0.56–22.68)	0.48 (0.27–0.87)	0.45 (0.24–0.85)
4	1.03 (0.19–5.66)	2.16 (0.28–16.41)	0.34 (0.17–0.64)	0.32 (0.16–0.65)
5	0.83 (0.10–6.83)	2.32 (0.32–16.82)	0.15 (0.08–0.28)	0.14 (0.07–0.29)
Positive screening result				
At least one	Reference	Reference	Reference	Reference
No	2.97 (1.27–6.94)	1.07 (0.43–2.70)	1.09 (0.72–1.67)	0.94 (0.59–1.48)

*Note*: Only statistically significant factors are shown in this table.

^a^ Adjusted for age, gender, education, income level, marriage, education, occupational exposure, prior lung disease, and prior screening number.

The prevalence of smoking relapse in baseline former smokers was 22.9%, which was significantly varied by variables such as age, smoking pack‐year, education level, occupational exposure, and prior screening frequency (Table 2). After adjusting for personal characteristics and screening history, we found that the relapse risk in baseline former smokers was significantly increased with the number of smoking pack‐years, household income, and prior screening number. Compared to those who had no occupational exposure, the odds of relapse were 13.47 (95% CI:6.71–27.04) in those with occupational exposure. Similar to smoking initiation in baseline never smokers, the relapse risk in baseline former smokers was also significantly reduced with age. However, no significant associations were found between prior screening frequency, positive screening results, and smoking relapse (Table 3).

In 887 baseline smokers, the point prevalence of smoking abstinence was 23.7% (210/887). Of 210 quitters, 167 were sustained abstinence, corresponding to an 18.8% sustained abstinence prevalence. Based on multivariable analysis result (Table 3), point smoking abstinence was associated with older age, female gender (adjusted OR = 7.21, 95% CI: 1.82–28.57), and prior lung disease (adjusted OR = 1.80, 95% CI: 1.05–3.08). In contrast, higher smoking pack years and income, and more prior screening numbers were the negative predictors of the prevalence of smoking abstinence. Compared to smokers who only received their baseline screening, the chance of smoking abstinence was reduced by over 80% in those who attended 5 rounds of screening (adjusted OR = 0.15, 95% CI: 0.08–0.27). The predictors for sustained smoking abstinence were similar to those for point abstinence.

Of 742 participants, who reported smoking in this survey, 740 reported their nicotine dependence and motivation to quit smoking. As shown in Table 4, over 60% and 90% of current smokers in the survey reported that they started smoking within 30 minutes after waking up and had not attempted or had no specific plan to quit, respectively. In addition, the proportion of >30 minutes of the first cigarette after waking up was gradually decreased with the increase of screening rounds (*p* = 0.013).

**TABLE 4:** Nicotine dependence and motivation in smokers at a survey

Item	Total	Number of prior lung cancer screening round						Prior positive screen results		
		1	2	3	4	5	*p*	No	Yes	*p*
Time of the first cigarette after waking up										
>30 min	248 (33.5)	15 (39.5)	39 (39.4)	67 (39.0)	38 (33.0)	89 (28.2)	0.013	195 (33.5)	53 (33.5)	0.498
6–30 min	174 (23.5)	7 (18.4)	20 (20.2)	28 (16.3)	22 (19.1)	97 (30.7)		142 (24.4)	32 (20.3)	
Within 5 min	318 (43.0)	16 (42.1)	40 (40.4)	77 (44.8)	55 (47.8)	130 (41.4)		245 (42.1)	73 (46.2)	
Plans to quit smoking										
No specific plan	697 (94.2)	37 (97.4)	92 (92.9)	162 (94.2)	108 (93.9)	298 (94.3)	0.908	545 (93.6)	152 (96.2)	0.223
Will quit within 1 year	43 (5.8)	1 (2.6)	7 (7.1)	10 (5.8)	7 (6.1)	18 (5.7)		37 (6.4)	6 (3.8)	

Of 210 participants who quitted after baseline, 120 (57.1%) provided their reasons for quitting smoking (Figure S1). The most common reason was ‘Smoking is harmful to health’ (75.8%), followed by ‘Objection from family members’ (39.2%) and ‘Affect the health of other people’ (31.7%), while only 20% and 3.3% of quitters reported prior positive screening results and economic burden as triggers for their smoke cessation, respectively.

## 4 DISCUSSION

In this multi‐centered study, after a maximum of 5 rounds of LDCT screening, a significant reduction in smoking prevalence was observed. This reduction resulted from the prevailing smoking abstinence in baseline current smokers over the relapse in baseline former smokers and smoking initiation in baseline non‐smokers. In addition, negative screening result was a predictor of smoking initiation in baseline non‐smokers, while the increase of screening rounds was associated with a decreasing likelihood of smoking abstinence in baseline current smokers.

The attendance of a lung cancer screening trial by itself might promote smoking cessation. Though the difference in smoking cessation rate between the screening arm and control arm was varied in randomized controlled trials of LDCT screening, all these trials demonstrated a significant overall higher smoking cessation rate when compared to the general population.^14^, ^16^, ^17^, ^25^, ^26^ Cohort studies also reported a high quit rate.^27^, ^28^, ^29^ Overall, the smoking cessation rates of baseline current smokers who quit during the study period ranged from 7% to 23%.^30^ Due to the deeply entrenched culture of smoking and insufficient tobacco control interventions, the prevalence of smoking remained high, while the quitting rate was as low as 11.0%.^31^ In this study, the 23.7% of smoking cessation rate was far higher than that of Chinese adult male smokers, which implied that lung cancer screening could also be used as a “teachable moment” for smoking behavior change in China, despite the prevailing misconceptions and social norms surrounding smoking.^32^ However, similar to those in the randomized controlled trials, selection bias may also exist in this cohort since the education and socioeconomic status of participants might be different from those who were invited but did not participate in the study.^33^

Previous screening results may also contribute to the change in smoking behavior. Firstly, positive screening results might increase the smoking cessation rate and decrease the smoking relapse rate.^13^, ^34^, ^35^ In the National lung cancer screening trial, any false positive screening result during 5 years of follow‐up was associated with subsequent significantly increased point and sustained abstinence among smokers.^34^ In the Danish lung cancer screening trial, a 1‐year quit rate was significantly associated with CT findings necessitating 3‐month repeat CT scans. However, this association became insignificant during all five screening rounds.^13^, ^25^ This suggests that the effect of a positive screening result on smoking cessation might be a transient, short‐term effect.^36^ Conversely, some other studies found no impact of screening outcome on future smoking abstinence.^36^, ^37^ Similarly, after adjusting for other factors, we did not find a significant smoking cessation effect of prior positive results as to both prevalence abstinence and sustained prevalence. Furthermore, more screening rounds were associated with a lower probability of smoking cessation as well as a higher nicotine dependence, which implied that besides “teachable moment,” to some extent lung cancer screening might also serve as a “license to smoke.” In this study, negative screening result was significantly associated with an increased risk of smoking initiation in baseline non‐smokers, which suggested that the negative results gave them a “license to smoke.” However, some other studies did not evidence the “license to smoke” effect of lung cancer screening.^14^, ^26^

Data of smoking relapse related to lung cancer screening were relatively limited. Relapse rates of baseline smokers who restarted smoking during the study period ranged from 1.6% to 12%.^30^ In a Danish trial, relapse rates of former smokers at 1‐year follow‐up were similar in CT and control arm (10% vs 10.5%), and remained stable across the following 5 years.^13^, ^25^ Lower relapse rate of CT and control arms were observed in the ITALUNG lung cancer screening trial, and no significant difference was observed between the two arms.^26^ The association between the screening results and smoking relapse was inconsistent. In the DLCST trial, the 1‐year relapse rate in baseline ex‐smokers with positive scans was significantly higher than those without no positive CT findings. However, this effect could not be found when all five screening rounds were considered.^13^, ^25^ In contrast, other studies did not find a relationship between screening results and relapse in long‐term former smokers.^34^, ^36^, ^38^ In this study, the prevalence of relapse in baseline former smokers was higher than those reported abroad, but, lower than the 33% of the general population in China.^39^ Moreover, no significant associations between the number of prior screening rounds, screening results, and smoking relapse in baseline former smokers were observed in this study.

Currently, most lung cancer screening guidelines recommend lung cancer screening for heavy smokers.^9^ However, less than 50% of incident lung cancers are among individuals who are eligible for screening.^40^ Risk prediction model which included other risk factors might improve the accuracy of screening eligibility criteria.^41^ In China's lung cancer screening guidelines, non‐smokers who had other risk factors were also recommended for lung cancer screening.^21^, ^42^ Thus, it is necessary to access the smoking behavior of never smokers in a lung cancer screening program. In this study, prior positive screening results were associated with a significantly decreased risk of smoking initiation in baseline non‐smokers, which suggested the effect of the “teachable moment” of lung cancer screening in non‐smokers.

Besides lung cancer screening, some other demographic predictors of smoking initiation, relapse, and abstinence in baseline non‐smokers, former smokers, and current smokers were also analyzed. In baseline non‐smokers, younger age significantly increased the risk of smoking initiation. Furthermore, it was also associated with an increased risk of relapse in baseline former smokers, and a decreased likelihood of smoking abstinence in baseline current smokers. The female gender was also associated with favorable smoking behavior as to smoking initiation and smoking abstinence. These results were also reported in previous studies.^15^, ^26^, ^35^ Compared to other workers, blue‐collar workers were more likely to smoke and were less successful in quitting.^43^ In this study, baseline former smokers with occupational exposure had a significantly increased risk of smoking abstinence as a subset of blue‐collar workers. In baseline current smokers, prior lung disease was associated with an increased likelihood of smoking abstinence, similar to another study.^44^ In addition, a high‐income level was correlated with a decreased likelihood of abstinence. This might be true since only 3.3% of quitters attributed their cessation to an economic issue, which was even lower than that of the general population in China.^45^

To our knowledge, this was the first study to evaluate the association between smoking initiation and lung cancer screening, and it was also the first report on the prevalence of smoking relapse, abstinence (point and sustained) in baseline former and current smokers in the context of lung cancer screening in China. However, several limitations should be noted. First, selection bias might exist, since participants enrolled in lung cancer screening likely had a heightened concern for their health and greater mortification to quit smoking. Second, the change in smoking behavior was attained retrospectively, the annual prevalence of smoking initiation, relapse and abstinence could not be accessed. Third, the smoking information in this survey was self‐reported and was not biologically validated. However, a prior study found that self‐reported smoking status was highly consistent with urinary/serum cotinine test results.^46^, ^47^ Besides, this study was just an ancillary survey for the LungSPRC project, thus self‐reported smoking status in this setting might be more reliable than in smoking cessation trials. Fourth, some potential cofounders were not included in the analysis. For example, nicotine dependence and motivation to quit information was not collected at baseline, while it was also reported as a predictor of smoking cessation.^13^, ^44^ Finally, because this was an ancillary study of a lung cancer screening program in rural China, data on some health issues was not obtained. As a result, no direct assessment of the effect of other smoking‐related health issues on smoking behavior was conducted in this study.

In conclusion, the study found that lung cancer screening had both “teachable moment” and “license to smoke” effects on smoking behavior in China, but there was an overwhelming effect of “teachable moment” on “license to smoke.” Therefore, smoking cessation should be integrated into lung cancer screening, and those at high risk of smoking initiation or relapse should be given special attention based on personal characteristics and prior screening history.

## AUTHOR CONTRIBUTIONS

YGF and FHZ had full access to all the data in the study and take responsibility for the integrity of the data and the accuracy of the data analysis. Conception and design: QHZ, YLQ; data collection: XBL, HW, ZWM, YL, HLP, YGF; analysis and interpretation: HL, YW, YGF, ZS; drafting the article: ZS, YGF; manuscript revision: YGF, QHZ, YLQ.

## FUNDING INFORMATION

This work was supported by the Chinese Central Government Public Health Special Subsidy. This study was also partly funded by the Cancer Foundation of China (grant number: CFC2020KYXM001 to YGF). Tianjin Natural Science Foundation (grant number: 18JCYBJC92100 to XBL), National Natural Science Foundation of China(grant number 81971650 to ZWM). Key R & D projects of Science and Technology Department of Sichuan(grant number 2020YFS0212 to HL), Tianjin Key Medical Discipline (Specialty) Construction Project (TJLCMS2021‐02 to YGF).

## CONFLICT OF INTEREST

None.

## ETHICS STATEMENT

This study received approval from the institutional review board of Tianjin Medical University General Hospital (2019–203‐01), and informed consent was obtained for each participant.

## Supporting information


Figure S1

## Data Availability

The data that support the findings of this study are not publicly available but available on request from the corresponding author.
