# Supplementary figures and images for: The impact of low‐dose CT on smoking behavior among non‐smokers, former‐smokers, and smokers: A population‐based screening cohort in rural China
Source: Cancer Med. 2022 Jul 27;12(4):4667–78. doi: 10.1002/cam4.5073 (PMC9972152; doi:10.1002/cam4.5073)

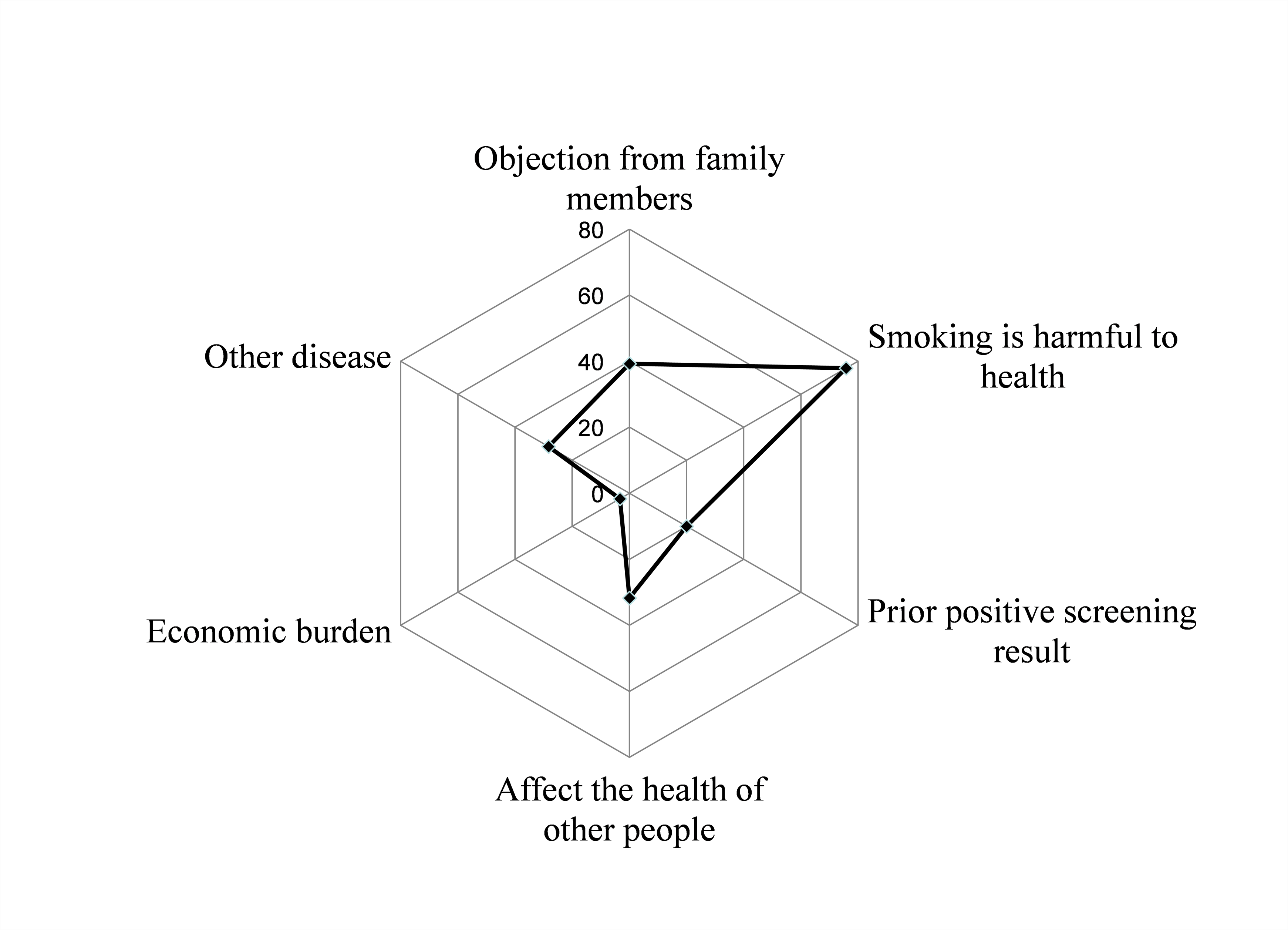

Supplement: Supplementary file 1 — Figure S1 [file CAM4-12-4667-s001.tif]
